# Supplementary material for: Biosensing Chlorpyrifos in Environmental Water Samples by a Newly Developed Carbon Nanoparticle-Based Indirect Lateral Flow Assay
Source: Biosensors (Basel). 2022 Sep 7;12(9):735. doi: 10.3390/bios12090735 (PMC9496275; doi:10.3390/bios12090735)
Supplement: Supplementary file 1 [file biosensors-12-00735-s001.zip › biosensors-1863095-supplementary.pdf]

Tables

Table S1. Collected surface water samples.

Sample No.	Code	Country of origin	Sample location	Sampling date
1	BP	Belgium	Botanic garden pond, Brussel	2019/12/30
2	OR	the Netherlands	Rhine river, Oosterbeek	2019/12/28
3	PS	France	Seine river, Paris	2019/12/23
4	RH	the Netherlands	Leuvehaven harbour, Rotterdam	2019/12/28
5	WP	the Netherlands	Campus pond, Wageningen	2022/04/15
6	WB	the Netherlands	Agricultural brook, Wageningen	2022/04/15
7	RA	Italy	Aquaponics farm, Rome	2021/04/12
8	WT	the Netherlands	Tap water, Wageningen	2022/04/15

Table S2. Average relative LFIA readings of all the fortified water samples LFIAs.

Sample[1]	200 ppb[2]		20 ppb		2 ppb		0 ppb	
	Mean	St.dev.[3]	Mean	St.dev.	Mean	St.dev.	Mean	St.dev.
BP	0.110	0.035	0.656	0.045	1.431	0.124	2.346	0.025
OR	0.122	0.026	0.552	0.021	1.437	0.139	2.146	0.247
PS	0.145	0.031	0.350	0.023	1.764	0.014	2.051	0.181
RH	0.140	0.015	0.629	0.052	1.433	0.015	2.621	0.363
WB	0.083	0.005	0.374	0.002	1.267	0.012	1.866	0.045
WP	0.173	0.098	0.428	0.005	1.615	0.064	1.959	0.201
RA	0.135	0.034	0.370	0.033	1.339	0.090	2.437	0.130
WT	0.263	0.064	0.493	0.001	1.402	0.008	2.392	0.005

[1] BP = Pond Brussels, OR = Rhine Oosterbeek, PS = Seine Paris, RH = Harbour Rotterdam, WB = Brook Wageningen, WP = Pond Wageningen, RA = aquaponics water, Rome and WT = tap water, Wageningen. [2] ppb = parts per billion (μg/L). [3] St.dev. is standard deviation (n=2).

Figures

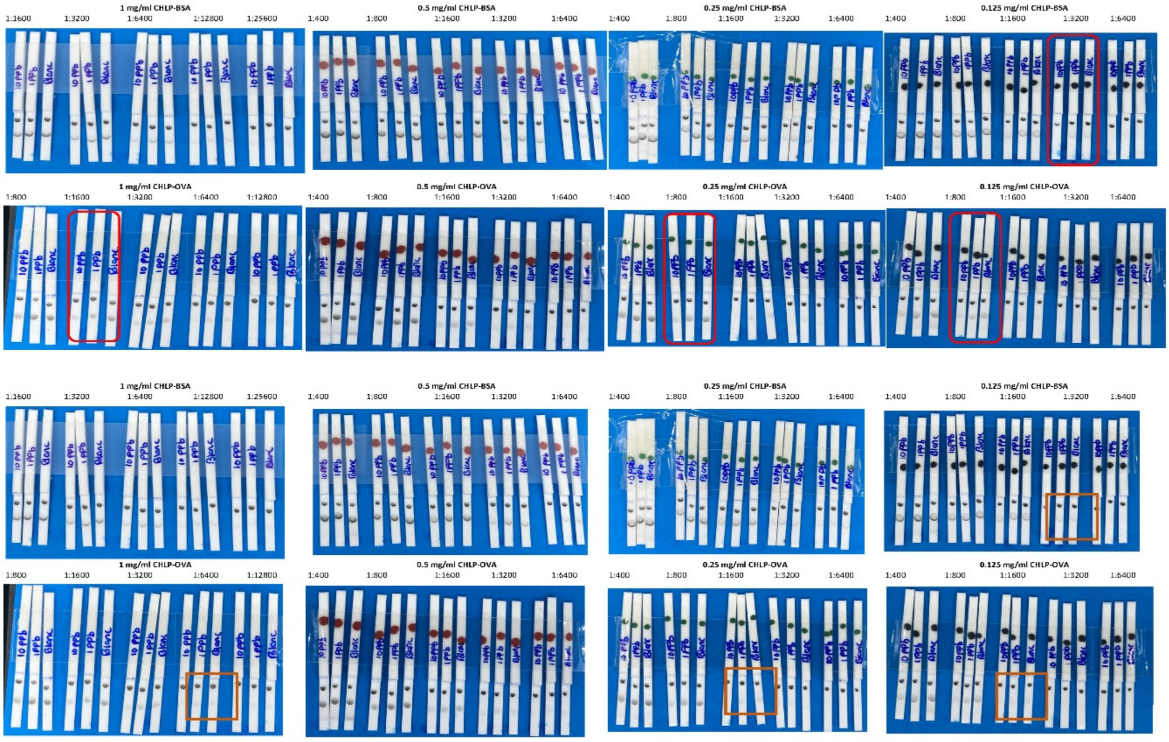

Figure S1. Method setup by the implementation of spot-based strips with constant concentrations of DAG pAb for the control spot (upper) and varying concentrations of CHLP-protein conjugates for the test spot (lower) on the membrane combined with serial CHLP-mAb dilutions.

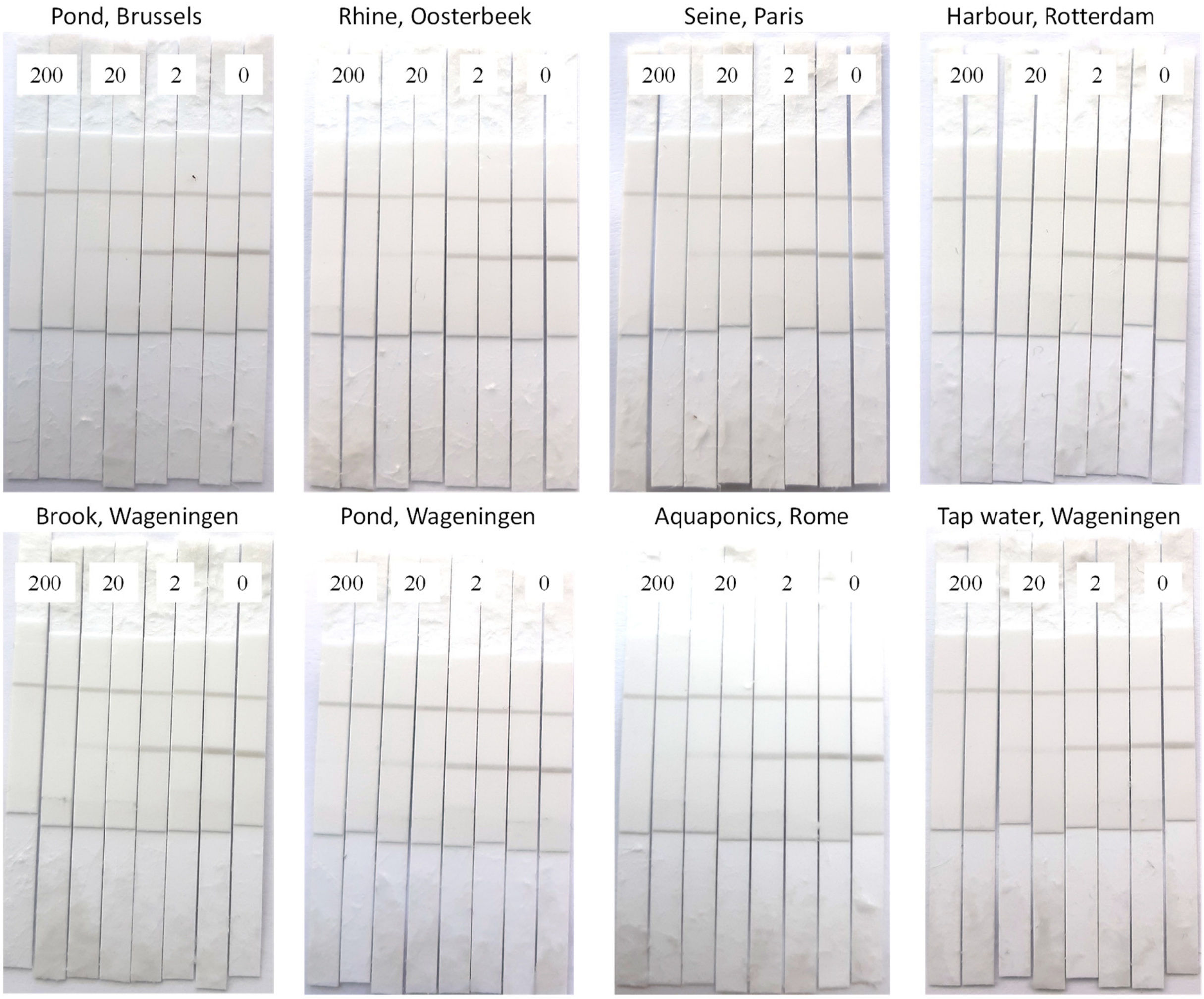

Figure S2. Overview of LFIA results for all the assessed environmental water samples. Each concentration (µg/mL) was tested in duplicate. .

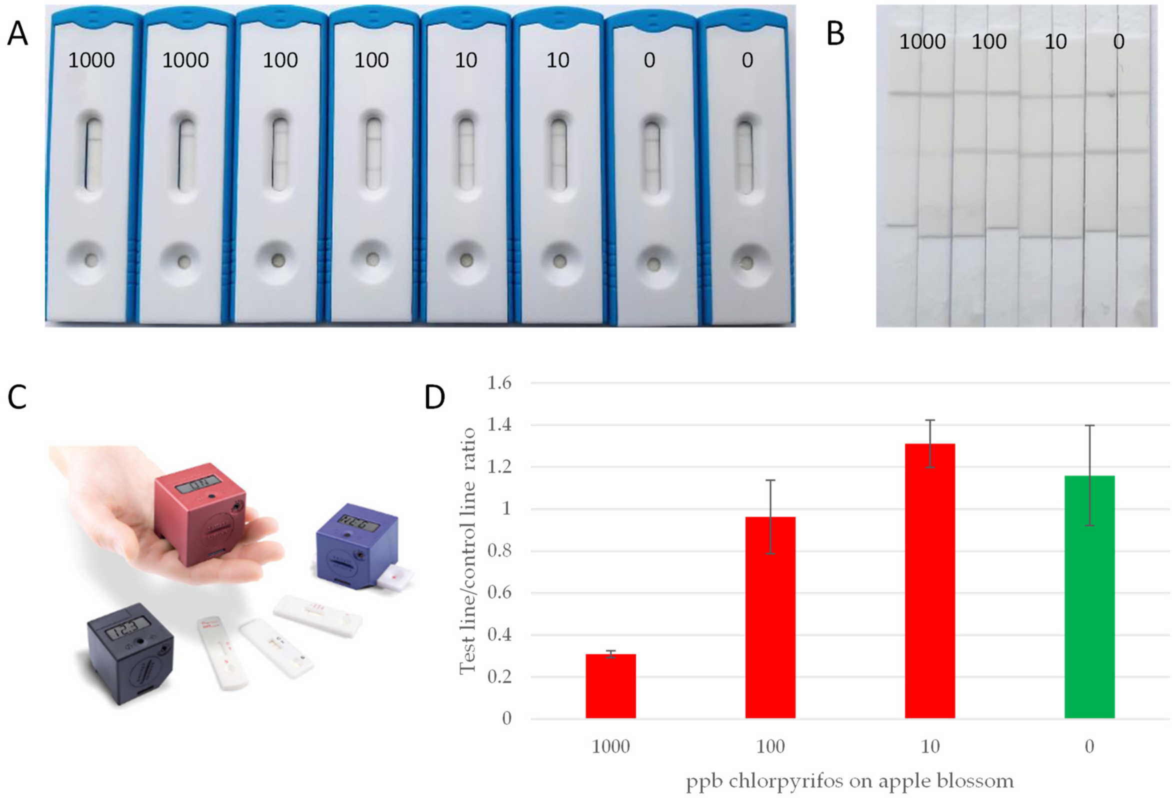

Figure S3. LFIA detection of chlorpyrifos in ppb (=µg/mL) (applied and dried) on apple blossom after water extractions by visual (A, B) readout and using a cube digital imager (C), for duplicate readings (D). The test line signals are divided by the control line signals to acquire T/C ratios.

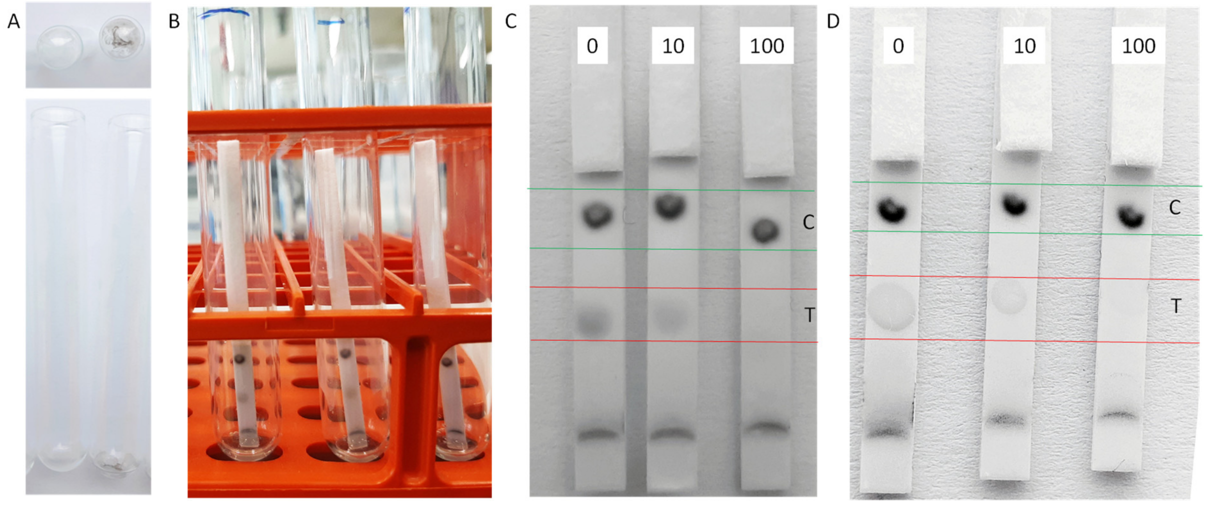

Figure S4. Simplification of the LFIA by drying assay reagents and buffer in tubes (A), after which a water sample containing chlorpyrifos is added and the LFIA inserted (B), showing 10 µg/mL sensitivities for spot-based LFIAs in duplicate measurements after visual readout (C, D). See the main body of the text for more explanation.
